# The multifunctional role of Notch signaling in multiple myeloma

**DOI:** 10.20517/2394-4722.2021.35

**Published:** 2021-04-14

**Authors:** Hayley M. Sabol, Jesus Delgado-Calle

**Affiliations:** 1 Department of Physiology and Cell Biology, University of Arkansas for Medical Sciences, Little Rock, AR 72205, USA.; 2 Winthrop P. Rockefeller Cancer Institute, University of Arkansas for Medical Sciences, Little Rock, AR 72205, USA.

**Keywords:** Notch, tumor microenvironment, multiple myeloma, bone, osteocytes, osteoclasts, osteoblasts, γ-secretase inhibitors

## Abstract

Multiple myeloma (MM) is a hematologic cancer characterized by uncontrolled growth of malignant plasma cells in the bone marrow and currently is incurable. The bone marrow microenvironment plays a critical role in MM. MM cells reside in specialized niches where they interact with multiple marrow cell types, transforming the bone/bone marrow compartment into an ideal microenvironment for the migration, proliferation, and survival of MM cells. In addition, MM cells interact with bone cells to stimulate bone destruction and promote the development of bone lesions that rarely heal. In this review, we discuss how Notch signals facilitate the communication between adjacent MM cells and between MM cells and bone/bone marrow cells and shape the microenvironment to favor MM progression and bone disease. We also address the potential and therapeutic approaches used to target Notch signaling in MM.

## INTRODUCTION

Multiple myeloma (MM) is a cancer that forms in the bone marrow due to the growth and accumulation of clonal, terminally differentiated B lymphocytes. Although considered rare, MM is the second most common hematological cancer, accounting for 10% of all hematological cancers. The precise etiology of MM has not yet been defined, but genetic, environmental, and microenvironmental components, as well as age, are considered important factors for disease development^[1]^. The clinical presentation of the disease includes the detection of high levels of paraproteins produced by MM cells^[2]^. Bone pain and fatigue are typical symptoms and frequently the main cause of initial consultation. Formal diagnosis of MM requires the detection of elevated monoclonal paraprotein levels, serum immunoglobulin free light chain ratio (FLCR) > 100, the presence of at least 10% myeloma cells in the bone marrow, advance imaging to detect focal bone disease, and the display of end-organ damage, often referred to as CRAB: hypercalcemia, renal failure, anemia, and bone lesions^[3,4]^. MM is commonly preceded by a precancerous, benign condition known as monoclonal gammopathy of undetermined significance (MGUS)^[5,6]^. MGUS is characterized by the presence of serum M paraproteins (less than 3 g/dL), clonal plasma cells in the bone marrow (less than 10%), and no other major MM symptoms^[5,6]^. MGUS has 3 different subtypes including: non-IgM MGUS, IgM MGUS, and light-chain MGUS. Non-IgM MGUS (more common) and light-chain MGUS can progress to MM, while IgM MGUS is commonly associated with B-cell lymphoproliferative disorders, but also can progress to MM. Patients with non-IgM MGUS and light-chain MGUS have a 1% risk of progression to MM per year^[5]^. Smoldering MM (SMM) is distinguished from MGUS by a higher risk of progression to MM^[4,7]^. SMM patients are asymptomatic, but have higher serum M paraproteins (greater than 3 g/dL) and bone marrow plasma cells (10%−60%) than MGUS patients. SSM patients have a 10% risk of developing MM per year in the first 5 years^[4,7]^. In later stages, MM can progress to an advanced disease stage called plasma cell leukemia (PCL), which is diagnosed when 20% of white blood cells are abnormal plasma cells. PCL, which can also occur *de novo*, without preceding MM, is a very aggressive form of MM and has a low survival rate^[8]^. If eligible, MM patients typically undergo chemotherapy and stem cell transplantation, leading to a remission phase of variable duration^[2]^. However, disease relapse is very common in MM, and is followed by a second line of therapy and remission phase^[2]^. This cycle continues until drugs are not able to stop MM progression^[2]^. Although recent advances have significantly improved overall patient survival, MM still remains incurable due to the high rate of relapse. Thus, new therapeutic approaches to treat MM progression and prevent disease relapse are desperately needed.

Another area where unmet medical needs remain is the management of the bone disease that accompanies MM. Approximately 80% of patients with MM present bone lesions, which can cause severe bone pain and pathological fractures in 60% of MM patients^[9,10]^. The skeletal complications have a major impact on patient morbidity and mortality, and decrease the quality of life of MM patients. Mechanistically, the growth of MM cells in the bone marrow disrupts bone homeostasis by increasing the number of bone resorbing osteoclasts, stimulating apoptosis of matrix embedded osteocytes, and decreasing the number and function of bone forming osteoblasts^[9–11]^. As a result, MM patients display exacerbated bone resorption and a concomitant suppression of bone formation that leads to the formation of focal osteolytic lesions, which weaken the bone and increase the risk of bone fractures^[9,10]^. Bisphosphonates, and more recently Denosumab, a neutralizing antibody against Rankl, are potent anti-resorptive agents and the mainstay treatment for MM-induced bone disease due to their ability to prevent bone loss and to minimize the risk of fractures^[10,12–14]^. However, bisphosphonates only stop the bone disease and do not repair damaged bone. Because the bone disease and fractures persist, even in patients in complete remission, new clinical interventions are necessary to repair and/or build new bone in MM patients. Despite the promising results seen with the use of bone-forming agents (anti-Sclerostin antibodies) in preclinical animal models^[15–17]^, no bone anabolic therapies have been approved for the treatment of MM yet.

MM is highly dependent on the bone marrow microenvironment^[18,19]^. In recent years, research efforts have focused on understanding the role of the MM tumor microenvironment in MM to identify new targets and develop novel therapeutic approaches. The inclusion of agents targeting the supportive effects of the marrow niche, including bortezomib, lenalidomide and other immunomodulatory drugs, has significantly changed treatment strategies in MM and improved patient outcomes. Importantly, some of these agents can have dual effects, reducing MM growth and mitigating the deleterious effects of MM cells on bone^[20,21]^. Notch signaling, a pathway mediating the communication between adjacent cells, has been identified as aberrantly activated in the MM tumor niche. In this manuscript, we review the last findings and pleiotropic effects of Notch signaling activation in MM and discuss new advances in the therapeutic strategies aiming to target Notch for the treatment of MM and the associated bone disease.

## THE NOTCH SIGNALING PATHWAY

**Figure 1.**
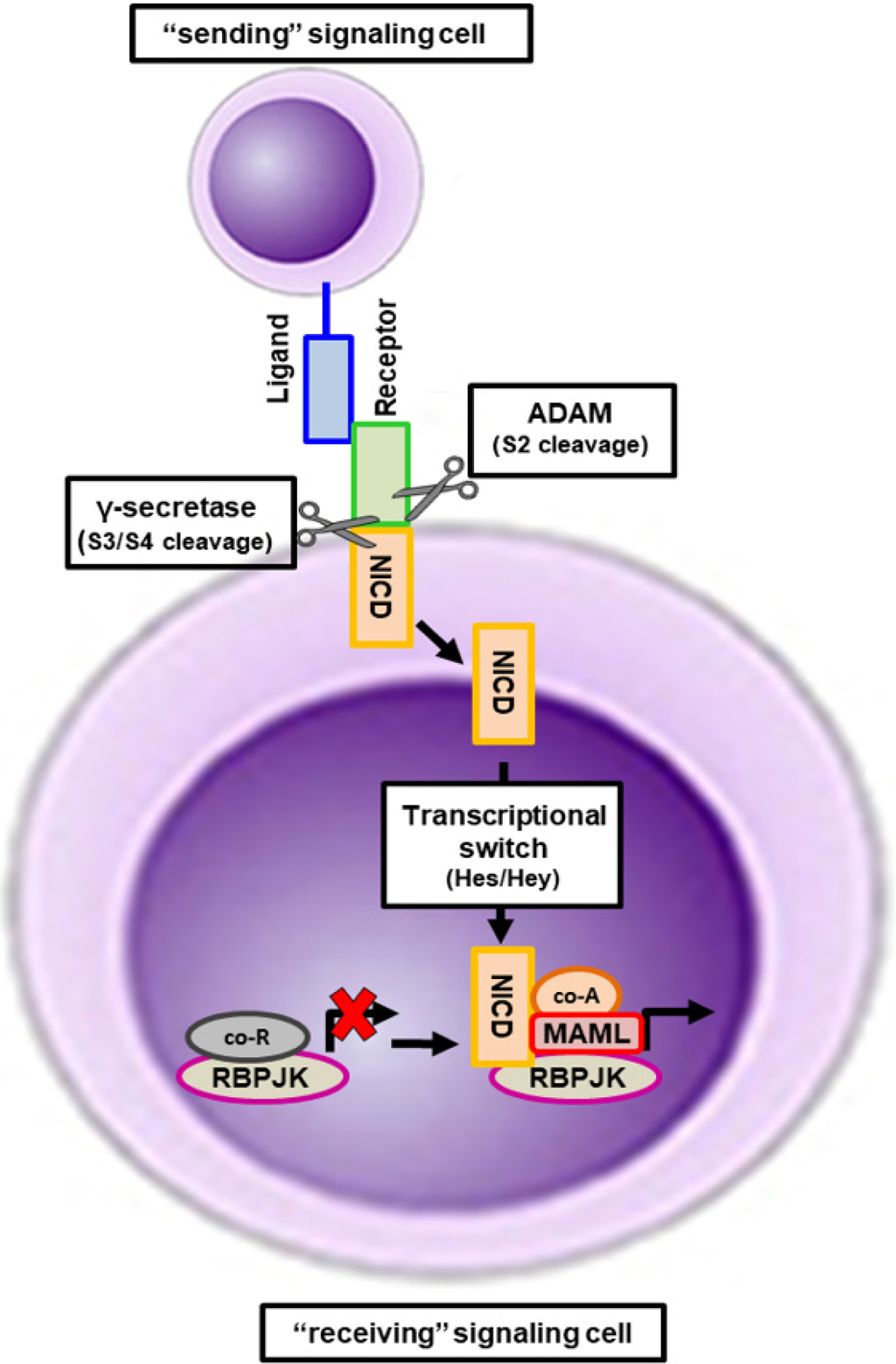
Activation of the canonical Notch signaling pathway. Notch mediates the transmission of near-range signals by physical contact between adjacent cells. The sending signal cell expresses Notch ligands that bind to Notch receptors in the receiving signal cell. Upon binding, the intracellular portion of the Notch receptor undergoes sequential proteolytic cleavage by Adam enzymes and the γ-secretase complex. The cleavage by the γ-secretase complex frees the Notch intracellular domain (NICD) from the transmembrane Notch domain and it translocates to the nucleus. In the nucleus, NICD promotes a transcriptional switch by binding to RbpjK and displacing of co-repressors and promoting the recruitment of MAML proteins and other transcriptional activators, activating the gene transcription of Notch target genes of the Hes and Hey families.

Notch is a highly conserved signaling pathway that mediates short-range, cell-to-cell communication [Figure 1]^[22–24]^. In fact, under most circumstances, Notch signaling transmission requires physical contact between cells. The Notch pathway core components include Notch transmembrane receptors (1–4), 2 families of membrane-bound ligands, Delta-like ligands (Dll) 1–4 and Serrate-like ligands (Jagged) 1 and 2, the Notch receptor proteases Adam and γ-secretase, and the nuclear effector Csl (also known as Cbf1 or RbpjK)^[22–25]^. The Notch membrane-bound ligands are expressed in signal-sending cells. Notch ligands are type I transmembrane proteins with 3 main structural domains: a N-terminal DSL motif, a specialized tandem EGF repeat called the DOS domain, and EGF-like repeats^[26,27]^. Both the DSL and the DOS domains are involved in receptor binding^[26,27]^. Notch receptors, expressed in the signal-receiving cell, are single-pass type I transmembrane proteins and can have both redundant and unique functions. The extracellular domain of Notch receptors is required for ligand binding^[28,29]^. Notch receptors do not have enzymatic activity and rely on a sequence of proteolytic cleavage events to become active^[30,31]^. When the Notch ligand and receptor come into contact, there is a conformation change in the extracellular portion of the Notch receptor, which unmasks the cleavage site 2 (S2). This allows for Adam metalloproteases, Adam10 and Adam17, to cleave the Notch receptor at the S2 cleavage site, the first step leading to its activation^[32]^. After S2 cleavage, the γ-secretase complex cleaves at site 3 (S3)^[32]^. This cleavage causes the separation of the C-terminal portion of the receptor, known as NICD, which is now free to translocate to the nucleus. In the absence of NICD, RbpjK is bound to co-repressor proteins to prevent transcription [Figure 1]. Once in the nucleus, the NICD displaces the co-repressors and interacts with RbpjK, as well as recruits mastermind-like (MAML) proteins to form a multi complex that induces transcription of various genes downstream^[33,34]^. Notch target genes include genes in the hairy and enhancer-of split (Hes) and hairy and enhancer-of-split with YRPW (Hey) families, which are helix-loop-helix proteins that function as transcriptional regulators^[35]^. This limited set of target genes is believed to mediate the diverse biological outcomes downstream the activation of Notch receptors.

Notch has pleiotropic biological functions, which are both context and cell dependent^[22,23,36]^. For instance, Notch signals mediate essential biological processes, including cell differentiation, apoptosis, proliferation, cell fate, and differentiation programs in both development and maintenance of adult tissues^[25]^. Given its relevant role in fundamental biological processes, aberrant Notch signaling underlies the pathophysiology of several human disorders^[24]^, including solid and hematological cancers^[36,37]^.

## DYSREGULATION OF NOTCH SIGNALING IN MULTIPLE MYELOMA

It is well documented that the Notch signaling pathway is deregulated in MM and preclinical data suggest it contributes to the progression of MM^[25,38,39]^. Analysis of immunostainings for Notch components in bone marrow biopsies from MM patients and healthy subjects revealed that the expression of Notch receptors 1 and 2 is increased in malignant plasma cells from MM patients compared to nonmalignant plasma cells or bone marrow from healthy individuals^[40–42]^. Notch receptor 3 expression is low in MM cells^[43]^. To the best of our knowledge, no studies have been conducted to determine if the expression of Notch receptor 3 is elevated in malignant plasma cells from MM patients or if it changes as the disease progresses from MGUS to PCL. Notch receptor 4 expression is low and in some cases undetectable in malignant plasma cells from MM patients and MM cell lines^[43]^. The expression of the Notch ligands Jagged 1 and 2, as well as the Notch target gene Hes5 has also been reported as elevated in MM patients^[40–42,44]^. Although some Notch components are aberrantly expressed in MM, the mechanism behind their dysregulation remains unclear. One possibility is that the increase in Notch members is due to genetic mutations. For instance, Notch receptor 2 increases have been associated with translocations t(14;16)(q32;q23) and t(14;20)(q32;q11)^[45]^. The increased copy number of Notch ligands and receptors has also been linked to trisomies of various chromosomes at which genes of the Notch pathway are located. This includes Notch1 (chr.9q34.3), Notch3 (19p13.2-p13.1), Dll3 (19q13), and Dll4 (15q14)^[25,44]^. *De novo* activation due to microenvironmental cues may also underlie the changes in the expression of Notch components. Supporting this notion, chromatin activation of genes at different steps of the Notch pathway, including ligands, receptor protease machinery, and downstream targets, has recently been detected in MM cells^[46]^. Moreover, preclinical studies from different groups have shown that interactions between MM cells and microenvironmental cells, such as stromal cells or osteocytes, can upregulate and/or change the repertoire of Notch receptors in MM cells^[43,47]^. Further studies are needed to determine the expression of Notch members in MM patients and changes that might occur as patients progress through the different stages of MM. Similarly, a better understanding of the underlying mechanisms involved in the transcriptional regulation of Notch components is required to determine the specific contribution of Notch dysregulation to MM disease.

### Notch and multiple myeloma tumor growth

Overexpression of Notch receptor 1, Jagged 1, and Jagged 2 occurs early in MM disease^[40–42]^. Additionally, dysregulation of Notch receptor 1 and Jagged 1 has been associated with progression from MGUS to MM^[41]^. These initial observations suggest that the main outcome of Notch activation in MM is increased tumor growth. Indeed, *in vitro* and animal studies show that both homotypic (among MM cells) and heterotypic (between MM cells and host cells) Notch activation increases MM cell proliferation and decreases apoptosis in both human and murine MM cell lines and primary cells from patients^[40,43,48–52]^. The increased levels of Notch ligands and receptors in MM cells facilitate physical communication with other neighboring MM cells, leading to increased Cyclin D1 expression and Il-6 production, which in turn stimulates proliferation and promotes survival^[43,53]^. The pro-survival effect of Notch is due to upregulation of anti-apoptotic proteins like Bcl-2 and Bcl-x2, and downregulation of Bax and Bak, pro-apoptotic proteins^[54,55]^. Notch signaling also appears to contribute to the migration of MM cells through the expression of the Cxcr4/Sdf1α axis system^[56]^. Yet, the specific contribution of individual Notch receptors to MM proliferation remains unclear. For instance, Notch receptor 1 overexpression increases MM cell proliferation, suggesting a relevant role of this receptor in MM growth^[57]^. We recently found that lentiviral-mediated inhibition of Notch receptor 3 reduces proliferation and Cyclin D1 expression in MM cells^[58]^. In contrast, genetic inhibition of Notch receptor 2 does affect the growth of MM cells^[58]^. Although yet to be determined, due to the low/undetectable levels of Notch receptor 4, it is likely that this receptor does not contribute to regulation of MM proliferation through homotypic interactions. Less is known about the specific contribution of Notch ligands to MM proliferation. In this regard, it has been shown that Jagged 2 regulates MM self-renewal *in vitro* and *in vivo*^[59]^.

Notch communication between MM cells and local microenvironmental cells also supports MM growth. Most of the work performed in this area has focused on the supportive role of stromal cells. Interaction of MM cells and bone marrow stromal cells induces the expression of Notch receptor 2 and Jagged 2 in MM cells, which results in increased expression of the Notch target genes Hes1, Hey2, and Hes5^[60]^. Stromal cells can activate Notch in MM cells via Dll 1 and cause an upregulation of Notch receptor 2 signaling, resulting in increased Notch transcription^[61,62]^. MM cells can also employ Notch ligands to activate the pathway in stromal cells, supporting the existence of bidirectional Notch communication between these cells types. In this regard, MM Jagged 2-mediated Notch activation stimulates Il-6, Vegf, and Igf expression in stromal cells, which in turn promotes MM growth and progression^[42]^. Osteoclast-MM communication via Notch also appears to promote MM survival through a mechanism involving the regulation of chrondoitin synthase 1 (Chys1) and Notch receptor 2 expression^[63]^. Our group recently demonstrated that osteocytes, the most abundant cells in bone^[11]^, activate Notch signaling in MM cells and increase MM cell proliferation by upregulating Cyclin D1^[43]^. In addition, osteocytes change the MM Notch receptor repertoire by rapidly increasing Notch receptor 3 expression and inducing the expression of Notch receptor 4^[43]^. Notch receptor 3 knockdown in MM cells partially inhibited osteocyte-induced MM proliferation^[58]^. Importantly, injection of MM cells with knockdown for Notch receptor 3 in mice resulted in smaller tumors^[58]^. In contrast, knockdown of Notch receptor 2 in MM cells did not impair the proliferative effects of osteocytes on MM cells^[58]^. These results suggest that osteocyte-MM interactions contribute to MM growth and are mediated by Notch receptor 3. However, it is likely that Notch receptor 1 and/or 4 also contributes to MM-osteocyte communication. These studies are among the first ones investigating the specific contribution of each Notch receptor to homotypic and heterotypic communication in MM. Future studies are needed to identify the Notch receptor-ligand binding requirements for the different interactions between MM cells and local microenvironmental cells.

γ-Secretase inhibitors (GSIs) are a class of small-molecule inhibitors that prevent the cleavage of γ-secretase substrates and block Notch signaling activation by precluding the cleavage of Notch receptors. GSIs are widely used to inhibit Notch and have been employed to better understand the contribution of Notch signaling to MM growth. Treatment with GSI decreases Notch signaling, which in turn causes MM cell apoptosis and decreases cell proliferation *in vitro*^[43,50,54,64]^. The Notch inhibition mediated by GSIs decreases Cyclin D1 expression, consequently increasing the portion of MM cells in the G_0_/G_1_ phase and decreasing those in the S-phase. In addition, pharmacological inhibition of Notch with GSIs decreases the expression of the anti-apoptotic protein Bcl-2, and activates Bak and Bax, resulting in activation of caspases and pro-apoptotic proteins, leading to increased MM cell death^[43,50,54,64]^. GSIs are also able to block the proliferative and pro-survival effects of heterotypic Notch activation by surrounding marrow cells. For instance, GSI blocked stromal Notch activation and decreased MM cell proliferation mediated by Jagged 2 overexpression^[47]^. Similarly, GSIs fully block osteocyte-induced Notch activation and increase proliferation in MM cells^[43]^. It is important to note that, besides Notch receptors, GSIs can also inhibit the processing of other gamma-secretase substrates, such as cell-surface receptors and proteins involved in embryonic development, hematopoiesis, cell adhesion, and cell/cell contact. Nonetheless, the results described above with GSIs are in line with those resulting from direct manipulation of Notch components in MM cells or environmental cells, and thus further support a role of Notch versus other γ-secretase substrates in MM cell proliferation.

In addition to GSI, other drugs have shown to achieve their anti-MM effects through the modulation of Notch signaling in MM cells. The ubiquitin specific peptidase 1 inhibitor SJB3–019A increases MM cell apoptosis by downregulating the expression of Notch receptors 1 and 2^[65]^. Further, Wang *et al*.^[66]^ demonstrated that treatment with sophocarpine triflorohydrazone (SCA), an alkaloid acting as an inhibitor of Notch receptor 3, decreased MM cell viability, activated apoptosis, and decreased Notch receptor 3 expression *in vitro*, in 2 different MM cell lines^[66]^. SCA treatment increased Bax proteins, decreased Bcl-2 proteins, and elevated caspase 3 levels^[66]^. Additionally, SCA treatment caused a decrease in the Notch target genes Hes1 and Hey1 expression^[66]^.

Together, the results discussed in this section highlight the contribution of Notch signals to MM progression by promoting cell cycle progression, improving survival in MM cells, and transmitting proliferative cues from cells in the marrow niche. However, it is important to note that MM proliferation is regulated by multiple signaling pathways^[19]^. Nonetheless, these collective findings provide the rationale for using Notch components as anti-myeloma therapeutic targets, an active area of investigation discussed in the “Notch components as therapeutic targets” section.

### Notch and drug-resistance

Relapse and refractory MM are hallmarks of MM disease, but the mechanisms underlying the development of drug resistance in MM are still under investigation. Disease relapse can be a consequence of the presence of drug-resistant MM clones initially present and/or emerging in the course of treatment^[67,68]^. MM refractory chemotherapy mechanisms are complex, and may involve a combination of genetic and epigenetic alterations, dysregulation of pathways involved in drug transport and cell death programs, and the presence of drug-resistant cancer stem cells or dormant cells^[67,68]^.

The focus of the research in drug-resistance has recently shifted towards the identification of protective mechanisms initiated by the marrow microenvironment^[67,68]^. Growing evidence supports an active role of Notch signals, particularly from stromal cells, in the acquisition of a drug-resistant phenotype in MM cells. Jagged 1-mediated activation of Notch signaling in MM cells by stromal cells is sufficient to protect MM cells from melphalan- and mitoxantrone-induced apoptosis^[69]^. This protective effect appears to be mediated by Notch receptor 1 and not by Notch receptor 2, as overexpression of Notch receptor 1 in MM cells prevents melphalan- and mitoxantrone-induced MM cell death^[69]^. Dll 1 signaling, through Notch receptor 2, has been shown to contribute to drug resistance to bortezomib, in both murine and human MM cells^[62]^. *In vitro* and *in vivo* studies demonstrated that both intrinsic and stroma-mediated drug resistance to bortezomib, lenalidomide, and melphalan also require Jagged ligands in MM cells^[70,71]^. Notch signals may also contribute to the development of resistance to mitoxantrone as inhibition of Notch signaling with GSI overcame the drug resistance to mitoxantrone induced by stromal cells^[50]^. Although in this case the ligand-receptor requirements were not evaluated, this effect appeared to be mediated by the transcriptional activity of the Notch target gene Hes1^[49]^. Notch signaling, in particular Notch receptor 1, is also required for resistance to doxorubicin. In this case, Notch receptor 1 regulates the expression of integrin αvβ5 in MM cells, which enhances MM cell adhesion to vitronectin^[72]^. Silencing Notch receptor 1 or blocking integrin αvβ5 with an antibody reduced MM cell adhesion to vitronectin and reverted in MM cells the protection from doxorubicin pro-apoptotic effects conferred by adhesion to vitronectin^[72]^. Importantly, blocking the Notch pathway with GSIs has been shown to prevent stroma-induced drug resistance by increasing sensitivity of MM cells to bortezomib, doxorubicin, and melphalan in cell cultures and animal models of MM^[50,62,69]^. Together, these studies suggest that specific Notch ligand-receptor signals confer resistance to different anti-MM drugs, an observation that could be exploited in the clinic to overcome drug resistance to particular therapies. Thus, the addition of Notch inhibitors to chemotherapy might be an interesting strategy to increase drug sensitivity in refractory MM patients. Nonetheless, animal and clinical studies are needed to determine the potential of targeting Notch inhibition to prevent or delay disease relapse in MM.

### Notch and angiogenesis

Angiogenesis, the process of new blood vessel formation from the existing vasculature, is necessary for the growth of MM cells^[73–75]^. Microvessel density is remarkably higher in the marrow of MM patients compared to those with MGUS or healthy subjects^[73–75]^. Neovessel density correlates with the disease stage and shrinks during remission, increasing again during the relapse/refractory phase, and reaching maximum expansion in PLC^[73–75]^. Elevated levels of marrow angiogenesis correlate with decreased MM patient survival^[76]^. Angiogenesis is regulated by a broad spectrum of locally produced factors, with Vegf (A-E) members being main drivers of this process in MM^[75]^. Vegf factors bind to Vegf receptors 1–3, activate endothelial cells, and initiate angiogenesis^[77]^. The bone marrow microenvironment in MM also facilitates angiogenesis because it is extremely hypoxic, which stimulates the production and release of Vegf.

Recent evidence suggests that Notch signals between MM cells, marrow cells, and endothelial cells can contribute to angiogenesis in MM. Endothelial cells from MM patients exhibit higher expression of Jagged 1 and 2, Notch receptors 1 and 2, and Notch target genes than endothelial cells from MGUS patients^[78,79]^. *In vitro* work has shown that Jagged-mediated signals from MM cells can increase angiogenesis by activating Notch and stimulating the release of Vegf in both endothelial cells and marrow stromal cells^[77]^. *In vitro*, genetic knockdown of Notch receptor 1/2 or blockade of Notch signaling with GSI decreased angiogenesis induced by MM cells. Further, GSI treatment reduced the secretion of pro-angiogenic cytokines in conditioned media and decreased angiogenesis in animal models of MM^[48,78,79]^. Until recently, MM cells or stromal cells have been considered the main source of angiogenic factors in the MM niche. In a recent study from our group, we examined if osteocytes contributed to the increased marrow vascular density in MM patients. We found that the number of Vegf-A positive osteocytes is significantly increased in bones bearing MM tumors and positively correlates with tumor vessel area^[80]^. Hypoxia and MM cells increased Vegf expression in osteocytes and increased the pro-angiogenic capacity of osteocytes^[80]^. Vegf-A knockdown in osteocytes completely blocked the increased endothelial activity induced by MM cells or hypoxia^[80]^. These results demonstrate that osteocytes are a source of Vegf-A, and potentially other pro-angiogenic factors, in bones infiltrated with MM cells. However, whether Vegf-A production by osteocytes is dependent on Notch remains to be determined. Despite the positive *in vitro* and *in vivo* results observed with pharmacological blockade of Vegf in MM models, inhibition of Vegf in the clinical setting has not been successful, likely due to the contribution of other pro-angiogenic factors to this phenomena^[75]^. Further studies are needed to clarify if Notch inhibition suppresses the production of other angiogenic factors besides Vegf, and if it could be an efficacious strategy to contain angiogenesis in MM patients.

### Notch and multiple myeloma-induced osteolytic bone disease

Bone is a very dynamic tissue, constantly being renewed by a lifelong process known as bone remodeling, where mature bone is removed from the skeleton and new bone tissue is formed^[81]^. This process is orchestrated by the osteocytes, which coordinate the coupled and balanced activity of osteoclasts, bone resorbing cells, and osteoblasts, bone forming cells^[81,82]^. Notch signals contribute to physiological bone remodeling^[83]^. However, the role of Notch in bone is complex and cell-dependent. The incomplete understanding of the role of Notch in adult bone biology stems from the use of genetic manipulations in mice, which result in alterations in skeletal development that inevitably affects the adult skeleton. The effects of Notch on osteoclasts are controversial, with findings reporting both inhibition and stimulation of osteoclast differentiation after Notch activation^[84–86]^. The effects of Notch in cells of the osteoblastic lineage are dependent on the differentiation stage^[87–91]^. In osteocytes, Notch receptor 1 genetic activation from birth results in inhibition of bone resorption due to Opg upregulation^[92]^. In contrast, conditional activation of Notch signaling in osteocytes in mature bones triggers bone formation^[93]^.

The growth of MM cells in the marrow markedly alters bone remodeling, uncoupling the activity of osteoclasts and osteoblasts, tilting the balance towards bone resorption^[9,94,95]^. The ability of MM cells to shape the marrow into a pro-resorptive environment is mediated by several signaling pathways, including Notch^[96]^. Notch signaling can regulate osteoclastogenesis by 2 different mechanisms: (1) regulating the expression of pro-osteoclastogenic cytokines in MM cells, and (2) mediating the communication between MM cells and microenvironmental cells that leads to pro-resorptive effects. MM cells are a source of cytokines that regulate osteoclast differentiation, including Rankl and M-Csf^[97]^. Notch signaling regulates Rankl expression in MM cells^[98]^. Supporting this notion, we recently found that genetic deletion of Notch receptors 2 and 3 in MM cells or treatment with GSI significantly decreases Rankl expression and impairs their ability to stimulate osteoclastogenesis^[58]^, Rankl expression in MM cells is also stimulated by stromal cells, an effect depending on Notch activation^[98]^. Additionally, MM cells can promote osteoclastogenesis by direct activating Notch in osteoclasts, via Jagged 1 and 2 ligands^[25,98]^. MM cells also activate Notch signaling in osteocytes, which are the major source of Rankl in adult bone^[99,100]^. Upon activation of Notch signaling by MM cells, osteocytes undergo apoptosis^[43]^, which in turn increases Rankl expression, decreases Opg production, and enhances the ability of osteocytes to recruit osteoclast precursors^[43]^.

The contribution of Notch signals to the protracted suppression of bone formation in MM is unclear. Osteoblast precursors isolated from MM patients exhibit increased Notch signaling and decreased osteogenic capacity compared to precursors derived from healthy subjects^[101,102]^. Interestingly, treatment with GSI restored Runx2 expression and the osteogenic capacity in osteoblasts precursors from MM patients *in vitro*, suggesting a potential role of Notch signaling in the suppression of new bone formation induced by MM cells^[101,102]^. Yet, studies manipulating Notch components in osteoblastic cells are required to establish the specific contribution of Notch signaling in MM-induced suppression of osteoblastogenesis. It is also possible that MM cells suppress osteoblasts indirectly, by acting on other cells in the marrow microenvironment. In this regard, our group has reported that MM cells deeply alter osteocyte biology in bones infiltrated with MM tumors^[43]^. For instance, MM cells increase the expression in osteocytes of critical regulators of bone remodeling, such as Sclerostin or Dkk-1. Genetic and pharmacologic inhibition of Sclerostin dramatically improves bone health in animal models of established disease, with no effects on tumor growth^[15–17]^. Although it has been shown that genetic manipulation of Notch components in bone cells can result in changes in Sclerostin production^[103]^, whether the increase in osteocyte-derived Sclerostin induced by MM cells is secondary to Notch activation remains to be determined. Importantly, pharmacologic inhibition of this pathway in animal models of MM results in decreased bone resorption and mitigation of the osteolytic disease, with no significant effects on bone formation^[49,54,101,104,105]^.

Collectively, these findings suggest that Notch signals contribute to the development of bone disease in MM, primarily through the generation of a microenvironment conducive to bone resorption and destruction. Further studies are needed to clarify the potential role of Notch signaling in osteoblasts suppression.

## NOTCH COMPONENTS AS THERAPEUTIC TARGETS

Several strategies have been tested to inhibit Notch^[106–108]^. However, due to the role of Notch signaling in the development and homeostasis of multiple tissues, the majority of approaches developed so far have led to undesirable, dose-limiting toxicities. For a detailed review of the existing clinical trials testing Notch inhibitors in cancer patients, refer to the recent manuscript by Moore *et al.*^[106]^. The most traditional and common approach to inhibit Notch is to prevent the proteolytic cleavage of the Notch receptors by blocking the γ-secretase complex with GSIs^[109,110]^. GSIs are attractive due to their ability to unselectively inhibit Notch signaling regardless of the ligand-Notch receptor involved. However, GSIs can lead to severe unwanted side-effects on tissues endogenously regulated by Notch, particularly the gut, where Notch inhibition causes secretory goblet cell metaplasia^[110,111]^. So far, the FDA has only approved, via Orphan Drug Designation and Fast Track Designation, the use of the GSIs Nirogacestat and AL101, for the treatment of desmoid tumors and Notch-mutant adenoid cystic carcinoma respectively, an important breakthrough in this field of research^[106,107]^. A more targeted approach to inhibit Notch is the use of monoclonal antibodies or soluble decoys against individual Notch components to disrupt specific ligand-receptor interactions. Several antibodies against Dll 4 (Demcizumab) and Notch receptors 1, 2, and 3 (Brontictuzumab and Tarextumab) have been tested in clinical trials^[106,107]^. Unfortunately, lack of clinic benefit over standard of care or intolerable toxicities have precluded the approval of these agents in the clinic. Moreover, these strategies are limited to particular Notch ligand-receptor interactions and thus, might not have a widespread application for the treatment of cancer patients. Another approach is the use of small-molecule inhibitors to target the Notch transcription complex (i.e., SAHM1, RIN1, and CB-103)^[106,107]^. Promising results have been obtained in preclinical models with these agents, particularly with CB-103, which exhibited a safe profile with no gut toxicity^[112]^. Ongoing clinical trials are evaluating CB-103’s anti-tumor efficacy in solid and hematological malignancies (NTC034226790).

To this date, no Notch inhibitors are approved for the treatment of MM. In fact, only 2 trials have tested Notch inhibition in MM patients. The effects of the GSI inhibitor RO4929097 were tested in MM patients after autologous stem cell transplantation (NCT01251172). Unfortunately, although no severe side-effects were reported in this study, the clinical trial was withdrawn due to the termination of drug development by the company. Recently, interest in Notch inhibitors has increased due to the observation that the γ-secretase complex cleaves BCMA from the membrane of MM cells, decreasing the amount of available target for chimeric antigen receptor (CAR) T cells specific for BCMA^[113]^. The anti-MM efficacy of BCMA-specific CAR T-cells in combination with GSI JSMD194 in relapsed or persistent MM patients is currently being tested in clinical trial NCT03502577. In this line, several companies have announced the testing of their GSIs (i.e., Nirogacestat and AL102) in combination with BCMA-specific CAR T-cells in MM patient populations.

Given its multifunctional role in MM, Notch still remains an attractive therapeutic target. However, Notch-related therapies have not gathered momentum due to the toxicities seen with current therapeutic strategies and the difficulty of targeting multiple Notch ligand-receptor interactions. Our group has taken a new direction to improve the therapeutic index of GSIs for the treatment of MM^[105]^. We have developed a novel bone-targeted GSI to bypass Notch inhibition in other tissues, particularly the gut. Preclinical results in animal models of human and murine MM showed that our bone-targeted GSI approach results in specific inhibition of Notch in skeletal tissues and decreases MM growth and bone destruction, without inducing gut toxicity. Ongoing pharmacokinetic and pharmacodynamics studies, as well as a full assessment of the safety profile, should provide a better picture of the potential of this new approach for the clinic.

## CONCLUSION

**Figure 2.**
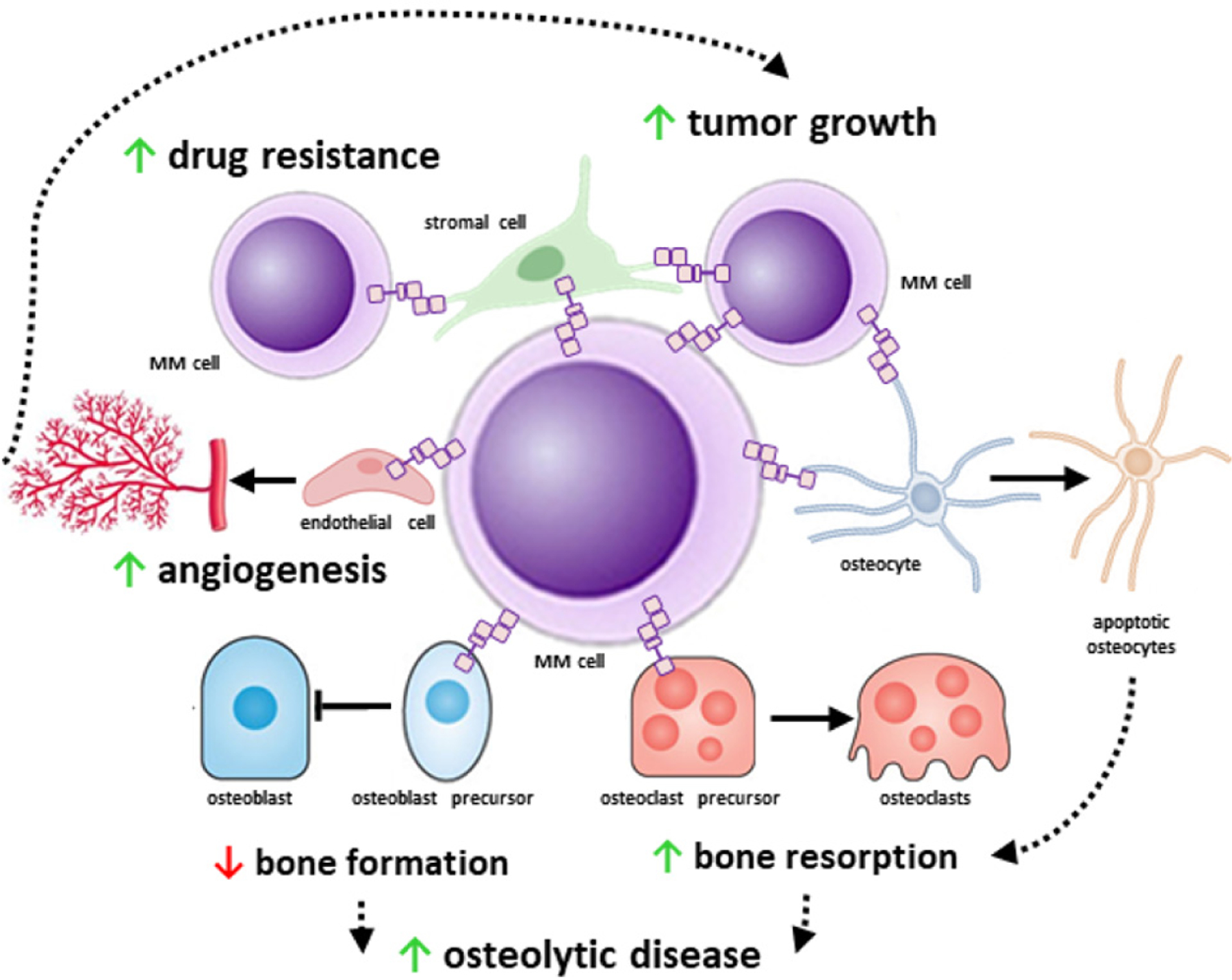
The multifunctional role of Notch signaling in multiple myeloma (MM). MM cells exhibit increased expression of Notch components that help them to receive and transmit near-range signals from and to adjacent cells. Homotypic Notch communication between MM cells increases MM cell proliferation. Tumor growth is further supported by Notch signals received by stromal cells and osteocytes. In addition, MM cells enhanced angiogenesis via sending pro-angiogenic Notch signals to endothelial cells, stromal cells, and osteocytes. The increased angiogenesis in turn aids tumor growth by providing nutrients to the tumor. Both homotypic and heterotypic (from stromal cells) Notch signals confer drug-resistance to MM cells and promote MM cell survival. Moreover, Notch signals from and to bone cells contribute to the progression of the MM-induced osteolytic bone disease.

Progress in medical research has improved our understanding of tumor biology and defined the impact of the microenvironment on cancer pathogenesis. This is particularly true for MM, where the bone/marrow niche plays a critical role in its onset and progression. MM cells locate in specialized niches in the marrow where they interact with stromal cells, endothelial cells, immune cells, osteoblasts, osteoclasts, adipocytes, and osteocytes. These interactions transform the marrow niche into an ideal environment for MM progression and the development of bone disease. Further, the marrow niche provides protection to drug-resistant MM cells, which can repopulate the marrow and induce disease relapse. Accumulating evidence supports that transmission of near-range signals via Notch between MM cells and marrow cells shapes the microenvironment and transforms it into a niche conducive to MM cell proliferation and survival, promoting drug resistance, and bone destruction [Figure 2].

Yet, several aspects of Notch signaling and its pleiotropic role of in MM remain to be resolved. A clear understanding of the specific role of each Notch component, the receptor-ligand specificity for homotypic and heterotypic interactions, and potential redundancies in receptor and ligand functions is required to identify effective and safer strategies to inhibit this pathway. It is expected that with the inclusion of next-generation RNA and DNA sequencing approaches in clinical practice, more information regarding the changes in expression and actionable mutations in Notch components will be available in the coming years. Particular attention should be paid to the changes during the progression from MGUS to MM to PCL in patients, as well as in the recurrence of the disease. Although Notch1 and 2 receptors are highly expressed in medullary and extramedullary MM cells^[40]^, whether Notch signaling plays a role in MM migration and extramedullary growth remains to be determined. Pharmacological inhibition of Notch with GSIs in preclinical models of MM shows promising dual strong anti-myeloma and anti-resorptive efficacy. Moreover, recent evidence shows that GSIs can enhance BCMA-directed CAR T-cell therapy by increasing the amount of surface BCMA target^[113]^. Unfortunately, the severe side-effects associated with the systemic inhibition of this pathway in other tissues have precluded the approval of GSI for the clinical care of MM patients. Although there are clinical trials evaluating the effects of targeting individual Notch components in other cancers, improved and safer strategies to target this pathway for the treatment of MM are still needed. Novel approaches to effectively and safely target Notch are currently under development to exploit this pathway in the clinic. In addition, more studies are required to determine if the combination of Notch inhibitors with other anti-MM therapeutics can delay/prevent disease relapse, repair damaged bone, and improve patient outcomes.
